# Humble Leadership and Employee Resilience: Exploring the Mediating Mechanism of Work-Related Promotion Focus and Perceived Insider Identity

**DOI:** 10.3389/fpsyg.2019.00673

**Published:** 2019-04-03

**Authors:** Yanhan Zhu, Shuwei Zhang, Yimo Shen

**Affiliations:** ^1^School of Political Science and Public Administration, Southwest University, Chongqing, China; ^2^Center for Chinese Public Administration Research, School of Government, Sun Yat-sen University, Guangzhou, China; ^3^School of Psychology, Southwest University, Chongqing, China

**Keywords:** humble leadership, employee resilience, work-related promotion focus, perceived insider identity, social information processing

## Abstract

Although the topic of employee resilience has recently received increased attention, existing research has largely failed to explore its situational triggers. Drawing on social information processing theory, the current study integrates the literature of humility and resilience to theorize the underlying mechanism through which humble leadership facilitates employee resilience. This research proposes a potential heterogeneous effect that humble leadership catalyzes employee resilience through multiple pathways. Field (*N* = 434) and experimental studies (*N* = 104) conducted in Mainland China support hypotheses that humble leadership enhances employee resilience through simultaneous increases in work-related promotion focus and perceived insider identity. Research implications are discussed, and directions for future research are offered.

## Introduction

Employee resilience is a capacity of employees that is supported and facilitated by organizations to positively cope, adapt, and even thrive in response to dynamic and challenging environments (Nguyen et al., 2016; Kuntz et al., 2017; Prayag, 2018). Luthans (2002, p. 702) defined it as the “developable capacity to rebound or bounce back from adversity, conflict, failure, or even positive events, progress, and increased responsibility.” Employee resilience has profound implications for promoting individual competence (Masten, 2001), enhancing individual responses to stressful circumstances (Youssef and Luthans, 2005), and improving job performance (Cooper et al., 2019). It also has emerged as a key capacity for employee growth and success when responding to challenges and/or inevitable adversity in the workplace (King et al., 2016). Furthermore, it has been stated that resilience should be regarded as an important source of competitive advantage beyond social and economic resources in organizations (Rego et al., 2016, 2017). More than simple adjustment, employee resilience embodies a transformational process (Näswall et al., 2015) in which employees tend to respond positively, persevere (Cooper et al., 2019), keep an open mind, and continuously improve in the ever-changing business world (Nilakant et al., 2014; Ou et al., 2015). Eventually, this leads to resilient employees assisting organizations in coping with increasing flux. Given the importance of employee resilience, how to activate it has become a very valuable and important issue. However, most prior research has focused on the outcomes of employee resilience and ignored the antecedent factors that bring it about (e.g., Ou et al., 2015; Cooper et al., 2019).

Leadership substantially influences the work lives of employees (Qian et al., 2018) and can be viewed as an important social context/situational factor that affects employee responses in the workplace (e.g., Williams et al., 2010; Nguyen et al., 2016; Wang et al., 2018a). Social context is “an integral ingredient enabling the kinds of mental models that lead to resilience” (Lengnick-Hall et al., 2011, p. 247; also see Cooper et al., 2019, p. 89). In the last decade, some researchers have given credit to the role leadership plays in the employee resilience-building process (e.g., Harland et al., 2005; Nguyen et al., 2016), suggesting that humble leadership be viewed as modeling how to grow, which can help employees embrace their own developmental journeys (Owens and Hekman, 2012; Rego et al., 2017). Unfortunately, however, this conceptualization has hitherto received scant academic scrutiny.

To address this issue, the current study sought to further elucidate employee resilience by examining its situational antecedents. Specifically, we complement the literature on resilience by testing whether humble leadership activates employee resilience. The impact of humble leadership on the development of others’ strengths has not received appropriate theoretical and empirical attention thus far (Rego et al., 2017). Second, in order to attain a comprehensive picture of the potential relationship above, we further decipher the path of how the employee resilience activation process may operate (Owens and Hekman, 2016). Our research proposes a potential heterogeneous effect, wherein humble leadership may catalyze employee resilience through multiple pathways, and provides empirical support for the argument proposed by Cooper et al. (2019), which states that employee resilience can be impacted by social processes.

**Figure 1:**
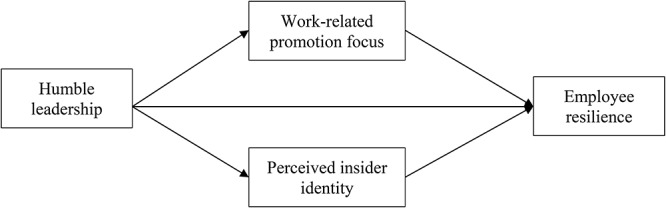
Theoretical model.

Social Information Processing theory (SIP; Salancik and Pfeffer, 1978) argues that the social environment provides cues that individuals may use to construct and interpret events. In accordance with this theory, we contend that humble leadership, as a bottom-up approach, may prime different aspects of intrinsic employee motivation, triggering behavior that follows. Perceived insider identity refers to “the extent to which an individual employee perceives him or herself as an insider within a particular organization” (Stamper and Masterson, 2002, p. 876; also see Schaubroeck et al., 2017). It provides a “reason to” type of motivation that encourages proactive employee reactions (Schaubroeck et al., 2017; Parker et al., 2010, p. 830). Work-related promotion focus involves striving to minimize the discrepancy between the actual and ideal states, being sensitive to the presence or absence of positive outcomes, and actively pursuing gains or advancement. It may provide a “can do” motivation, leading to resilient reactions in the workplace. The theoretical model, shown in Figure 1, unpacks the process of how humble leadership operates through work-related promotion focus and perceived insider identity to promote employee resilience. As employee resilience reflects a capacity that can be developed (Robertson et al., 2015; Kuntz et al., 2016, 2017), a deeper understanding of the situational factors that can induce resilience could deepen our understanding of this adaptive construct and lend practical insight into how to develop and manage it within organizations (Rego et al., 2016, 2017).

## Theory and Hypotheses

### Humble Leadership and Employee Resilience

Humble leadership is defined as a leadership style in which a leader evaluates him/herself and subordinates through a multifaceted and objective lens, appreciating subordinates’ positive worth, strengths, and contributions (Owens et al., 2013; Yuan et al., 2018). It contains three behavioral components: (a) a willingness to acknowledge one’s limits and mistakes; (b) shining a spotlight on employees’ contributions and strengths; and (c) keeping openness to advice, ideas, and feedback (Owens et al., 2013; Owens and Hekman, 2016). According to SIP theory, employees understand their work environments through the processing of social cues, which in turn shapes their reactions (Salancik and Pfeffer, 1978; Rego et al., 2017). Because of their high status, leaders can be viewed as vital social cues in workplaces (e.g., Yaffe and Kark, 2011). The actions of a leader in response to environmental challenges or adversities play a vital role in affecting employee resilience (Bullough et al., 2014). SIP theory also states that humble leadership represents powerful and valuable social information that can shape the perceptions of employees and influence employees’ reactions through the use of language and symbols. Humble leadership views problems and past mistakes as opportunities. By converting crises into developmental challenges, humble leadership provides intellectual stimulation to facilitate employees’ adaptive coping reactions (Owens et al., 2013). Additionally, humble leadership fosters supportive organizational contexts, including an empowering climate (Ou et al., 2014), legitimization of subordinate growth and development (Owens and Hekman, 2012), and reinforcing employee learning. It meshes closely with the concept of resilience, which, as noted earlier, consistently emphasizes positive coping and achieving growth. Moreover, humble leadership opens lines of communication (Elrod, 2013), increases employees’ psychological safety (Walters and Diab, 2016), and builds trust within organization (Elrod, 2013; Cooper et al., 2019), which can all be viewed as important antecedents to employee resilience (Cooper et al., 2019). Taken together, we predicted the following hypothesis:

Hypothesis 1 (H1): Humble leadership is positively associated with employee resilience.

### Mediating Roles of Work-Related Promotion Focus and Perceived Insider Identity

Work-related promotion focus influences employees’ approach and drive for desired work-related outcomes (Wallace and Chen, 2006). Employees exhibiting a promotion focus are inclined to attain advancement and minimize discrepancies between actual and desired end-states (Neubert et al., 2008). Compared to chronic promotion focus, work-related promotion focus has greater explanatory power for individual work reactions (Lanaj et al., 2012; Akhtar and Lee, 2014) and is more sensitive to priming by workplace contextual cues (Wallace and Chen, 2006; Kark and Van Dijk, 2007). Leadership can be viewed as a particularly salient contextual stimulus that is likely to evoke a specific self-regulatory focus in the minds of employees (Brockner et al., 2004; Graham et al., 2015; Wallace and Chen, 2006; Yang et al., 2018).

Humble leadership is perceived by employees as a model of how to grow and leads employees to feel that their own growth and improvement processes are legitimate and necessary (Owens and Hekman, 2012, 2016). Furthermore, it prompts employees to approach opportunities (Rietzschel, 2011) and orients employees to progressively strive toward achieving their potential. Additionally, through recognition of one’s own limits and past mistakes, humble leadership legalizes uncertainty, inspires employees’ growth, and creates climates of empowerment and autonomy (Ou et al., 2014; Yuan et al., 2018). This self-disclosure suggests that limits, past mistakes, and even setbacks can be overcome, ultimately facilitating development, shaping employee work-related promotion focus (Wang et al., 2018c) rather than work-related prevention focus which concerns about security and losses (e.g., Higgins, 2000; Akhtar and Lee, 2014). When employees exhibit a work-related promotion focus, more effective commitment and work engagement occur (Akhtar and Lee, 2014). A work-related promotion focus places emphasis on achievement, ideals, and gains (Akhtar and Lee, 2014), acting as a “can do” motivation to overcome work-related obstacles and impediments. Wallace and Chen (2006) argued that work-related promotion focus motivates employees to exert additional effort to succeed, which in turn may facilitate greater employee resilience. Hence, we predict the following hypothesis:

Hypothesis 2 (H2): Employee work-related promotion focus mediates the relationship between humble leadership and employee resilience.

Perceived insider identity is critical to viewing one’s organization membership in a favorable light (Schaubroeck et al., 2017). Lapalme et al. (2009) proposed that, as the organization’s agent, a leader plays an important role in employees’ perceived inclusion. Treatment by the leader facilitates the shaping of employees’ perceptions of social status within the organization (Lapalme et al., 2009) and then further influences employees’ subsequent workplace reactions (Tyler and Blader, 2000). Perceived insider identity is a product of employee’s cognitive processes, derived from social interactions, such as high-quality leader-subordinate relationships (Schaubroeck et al., 2017). It is likely to be influenced by leadership as social information concerning how leadership should be interpreted (Schaubroeck et al., 2017). Leadership is important to employees’ motivation and psychological connectedness to an organization (Schaubroeck et al., 2017). As Lapalme et al. (2009) discussed, perceived insider identity is associated with levels of leader support perceived by employees. Humble leadership fosters a supportive organizational context (Owens and Hekman, 2012; Yuan et al., 2018) and correlates with higher levels of information sharing and perceived psychological safety (Hu et al., 2018). As such, employees are more likely to feel supported and easily perceive themselves as insiders in an organization. According to the principle of reciprocity (Gouldner, 1960) and social exchange theory (SET; Blau, 1964), these employees would then feel that they should contribute more effort to the organization and are more likely to engage in resilient reactions to workplace adversity. Thus, we proposed that perceived insider identity is a conduit through which humble leadership influences employee resilience. We hypothesized the following:

Hypothesis 3 (H3): Perceived insider identity mediates the relationship between humble leadership and employee resilience.

## Materials and Methods

### Design of Studies

We carried out two studies to test our theoretical model. In Study 1, a field study was conducted in which survey data were collected from Mainland China. In this study, all scales adopted were originally written in English and then translated into Chinese following back-to-back translation (Brislin, 1970, 1986) protocols to ensure items accurately captured their original English meaning and were understood in Chinese. In order to establish internal validity between humble leadership and employee resilience, Study 2 examined hypotheses through a vignette-based humble leadership experiment (Rego et al., 2017). This method “enhances experimental realism and also allows researchers to manipulate and control independent variables, thereby simultaneously enhancing both internal and external validity” (Aguinis and Bradley, 2014: p.352; also see, Rego et al., 2017). Following the suggestion of Antonakis et al. (2016) that work sample tests and role playing can be useful to operationalize leadership in the workplace, we utilized both of these methods in our sample.

### Ethics Statement

All procedures performed were in accordance with the ethical standards of the Research Committee on Human Experimentation and with the Helsinki Declaration of 1964, as revised in 2000. In addition, our studies were approved by the Southwest University Ethics Review Board. All participants provided written informed consent, and all responses were anonymous.

## Study 1: Field Study

### Participants and Procedures

The data in Study 1 were collected from full-time working individuals through an exponential, non-discriminative “snowball sampling” method (Dudovskiy, 2016) in Mainland China. We invited dozens of full-time staff to participate in an anonymous survey. After they finished the questionnaires, we asked them to share this survey with their friends, relatives, and co-workers. After one month, a total of 478 participants responded to the survey. After list-wise deletion, the effective sample contained 434 staff (out of 478), resulting in a 90.8% response rate. Of these valid samples, 55.1% were female, and 66.8% were married. In terms of level of education, 21.5% completed junior college education; 42.6% possessed a bachelor’s degree; 21.2% had a master’s degree; and 14.7% had a doctorate. The average age was 34.1 years. In terms of tenure distribution, 19.8% reported less than 3 years; 28.6% were between 3 and 5 years; 28.1% were between 6 and 10 years; and 23.5% were 11 or more years.

### Measures

#### Humble Leadership

Humble leadership was measured with a 9-item questionnaire in which participants rated each item from 1 (*strongly disagree*) to 5 (*strongly agree*). We adopted a scale developed by Owens et al. (2013). Sample items included “My leader actively seeks feedback, even if it is critical” and “My leader shows appreciation for the unique contributions of others.” The estimated reliability of this measure was 0.90 in our study.

#### Employee Resilience

Employee resilience was measured with a 9-item questionnaire in which participants rated each item from 1 (*never*) to 7 (*almost always*). We adopted the scale developed by Näswall et al. (2015). Sample items included “I use change at work as an opportunity for growth” and “I re-evaluate my performance and continually improve the way I do my work.” The estimated reliability of this measure was 0.85 in our study.

#### Work-Related Promotion Focus

Work-related promotion focus was measured with a 9-item questionnaire. Respondents provided their agreement with each item from 1 (*strongly disagree*) to 5 (*strongly agree*). We adopted the scales developed by Wallace and Chen (2006) and Wallace et al. (2009). Sample items included “I take chances at work to maximize my goals for advancement” and “I spend a great deal of time envisioning how to fulfill my aspirations.” The estimated reliability of this measure was 0.79 in our study.

#### Perceived Insider Identity

Perceived insider identity was measured with a 6-item questionnaire in which participants rated each item on a scale from 1 (*strongly disagree*) to 5 (*strongly agree*). We adopted the scale developed by Stamper and Masterson (2002). Sample items included “I feel very much a part of my work organization” and “My work organization makes me believe that I am included in it.” The estimated reliability of this measure was 0.88 in our study.

### Results

#### Preliminary Analyses

Data were collected via a survey. To address the potential of common method variance (CMV), we ran a set of confirmatory factor analyses in Mplus 7.2 to examine the distinctiveness among the measures for variables employed in this study and to assess the severity of CMV (Podsakoff and Organ, 1986; also see Wei et al., 2015). If the CMV is a significant problem, then a single-factor model is as good as a full-factor measurement model (see Zhang et al., 2015), as these two models do not have a statistically significant difference. The hypothesized 4-factor model was selected as the best fitting model to the data (Table 1). The results suggested that CMV was unlikely to be a serious problem in this study. The means, standard deviations, and correlational coefficients of the variables adopted are shown in Table 2.

**Table 1:** Comparison of alternative path models.

Model test	χ^2^	df	χ^2^/df	CFI	TLI	RMSEA	SRMR
4-factor	1142.19	472	2.42	0.90	0.90	0.05	0.06
3-factor	2478.45	492	5.04	0.70	0.68	0.10	0.10
2-factor	3201.9	494	6.51	0.60	0.56	0.11	0.10
1-factor	3868.75	495	7.82	0.49	0.46	0.13	0.17

**Table 2:** Means, standard deviations, and correlations for the variables (study 1).

Variables	Mean	SD	1	2	3	4	5	6	7	8	
1. Gender	1.55	0.50									
2. Marriage	1.33	0.47	0.15**								
3. Age	34.07	6.58	-0.14**	-0.42**							
4. Education	3.24	1.06	-0.04	-0.01	-0.02						
5. Tenure	2.55	1.06	0.02	-0.29**	0.58**	0.05					
6. HL	3.61	0.77	0.15**	0.19**	-0.21**	-0.27**	-0.16**	(0.90)			
7. WPF	3.62	0.56	-0.02	0.05	-0.08	-0.14**	-0.16**	0.32**	(0.79)		
8. II	3.74	0.82	0.05	0.12*	-0.04	-0.12**	0.04	0.51**	0.16**	(0.88)	
9. ER	4.90	0.78	-0.01	0.05	-0.06	-0.03	-0.04	0.40**	0.39**	0.35**	(0.85)

*N = 434. Gender, marriage, age, education, and tenure are all the dummy coded variables (Gender: female = 2, male = 1; Marriage: unmarried = 2, married = 1; Education: complete high school or a lesser qualification = 1, complete junior college = 2, have a bachelor’s degree = 3, possess a master’s degree = 4, and have a doctorate = 5; Tenure: less than 2 years = 1, 2 to 5 years = 2, 6 to 10 years = 3, and 11 or more years = 4); HL, Humble Leadership; WPF, Work Promotion Focus; II, Perceived Insider Identity; ER, Employee Resilience. Cronbach’s alpha values shown in parentheses along the diagonal. ^∗∗^*p* < 0.01, ^∗^*p* < 0.05*.

#### Hypotheses Test

Considering the nature of our data, we tested all the hypotheses with Mplus 7.2. We adopted the SEM approach, as it allows simultaneous estimation of multiple indirect paths. To test the main effect, we modeled humble leadership as the predictor and employee resilience as the outcome and then conducted a regression analysis. The direct-path model provided the results for H1, which predicted that there was a significantly positive relationship between humble leadership and employee resilience (β = 0.42, *p* < 0.001; *R*^2^ = 0.18, *p* < 0.001) at the 95% confidence interval. Therefore, H1 was supported. Hypotheses 2 and 3 predicted mediating roles of work-related promotion focus (H2) and perceived insider identity (H3) between humble leadership and employee resilience. Using a bias-corrected confidence interval method, we applied bootstrapping (1000 samples) to test the indirect effects. The results support both the indirect effects of work-related promotion focus (indirect effect = 0.14, CI 99%, [0.06, 0.25], *p* < 0.001) and perceived insider identity (indirect effect = 0.11, CI 99%, [0.01, 0.28], *p* < 0.05). Therefore, H2 and H3 were supported. All of these effects (direct and indirect) are presented in Table 3.

**Table 3:** Standardized direct path coefficients of the hypothesized model (study 1).

	Path	Estimate	SE		
H1	HL-ER	0.42***	0.05		
**Bootstrap results for indirect effects**		
	**Path**	**Estimate**	**SE**	**LL99%CI**	**UL99%CI**

H2	HL-WPF-ER	0.14***	0.04	0.06	0.25
				**LL90%CI**	**UL90%CI**
H3	HL-II-ER	0.11*	0.05	0.01	0.28

*N = 434. Bootstrap sample size = 1000. ^∗∗∗^*p* < 0.001, ^∗^*p* < 0.05*.

## Study 2: Experimental Study

### Participants and Procedures

We used an experimental design to investigate the potential relationship between humble leadership and employee resilience (H1), as well as the mediating roles of work-related promotion focus (H2) and perceived insider identity (H3). Participants in the present study were full-time staff working in Mainland China. Participation was voluntary, and confidentiality was guaranteed. In the first round, 104 participants, who were enrolled in a Master of Public Administration Program (part-time) in a large university in the southwestern region of China, were invited to participate in our study. These individuals were then randomly assigned to one of the two conditions. These two conditions represented two different humble leadership levels. Four participants were omitted from analysis due to a substantial amount of missing data (making the valid response rate of 96%). To make sure cell sizes for the two conditions were equal (balanced design), we then invited another four individuals to participate by using the “snowball sampling” method. All of them were compensated with prepaid mobile phone cards value of RMB 30 Yuan (approximately 4.35 US dollars) for their participation. All participants held a bachelor’s degree or higher, 51% were female, and the mean age was 30.25 years (SD = 3.59).

### Manipulation and Measure

We created scenarios that resembled real encounters in workplace contexts. Following seminal works in the field of humble leadership (e.g., Owens et al., 2013, Owens and Hekman, 2016) that generally contend that a leader’s behaviors form the basis for employees’ attribution of humility, we deployed a behavioral approach to manipulate different levels of humble leadership. Initial versions of our scenarios were sent to two full-time staff of organizations for feedback on the realism and clarity. Minor revisions were made based on their feedback. We manipulated humble leadership in scenarios, resulting in a 2 (humble leadership: non-humble vs. humble) × 1 (employee resilience) factorial design. Condition 1, namely, the non-humble leadership condition, was the control group. Condition 2, namely, the humble leadership condition, was the experimental group. Participants were randomly assigned to one of the two conditions: non-humble leadership (*n* = 52) and humble leadership (*n* = 52). Participants received all materials in paper-and-pencil form. Participants were asked to put themselves into the situation described and were told that they needed to make their own decisions. To ensure the reliability and confidentiality of the participants’ responses, all were told that there were no right or wrong answers and that all information was given anonymously and would only be used for research purposes. The manipulation of humble leadership was achieved by inserting a vignette characterizing the leader as behaving in a humble way. We created the scenarios based on the seminal definition and items of humble leadership proposed by Owens et al. (2013). We used transactional leadership (van Dierendonck et al., 2014) in the control group as an example of non-humble leadership, because of its neutrality in terms of humility (Rego et al., 2017). Similar manipulations of humble leadership have been used in prior research (Owens and Hekman, 2016; Rego et al., 2017).

### Manipulation Check

We did a twofold manipulation check to control for alternative explanations of our results. First, we used a panel of two university faculty members who were experts in the domain of leadership to assess the definition of leadership versions of our scenarios. Both experts rated the humble leadership version as a case of very intense humility and rated the control group version as an example of which the leader does not show any qualities reminiscent of humble leadership. In addition, participants were asked to respond to a manipulation check: “I would characterize the leader as a humble leader” (from 1 = *strongly disagree* to 7 = *strongly agree*). As a result, the humble leadership manipulation was considered successful.

### Measures

Participants were required to “assume the part” of the employee in each vignette they read. They were then asked to complete a serial scale, which was used to reflect their likely reactions if they were to encounter the situations described. Participants reported the extent to which they considered the leader to be humble on a 7-point Likert scale (1 = *strongly disagree* to 7 = *strongly agree*). Other measures adopted were well-established scales. We adopted the 9-item scale used by Wallace et al. (2009) to assess work-related promotion focus. Cronbach’s alpha in this study was 0.79. Perceived insider identity was tested by a 6-item scale presented by Stamper and Masterson (2002). Cronbach’s alpha in this study was 0.88. Employee resilience was assessed using a 9-item scale developed by Näswall et al. (2015). Cronbach’s alpha in this study was 0.87.

### Results

The manipulation check showed that participants in the humble leadership group reported the leader as humbler than those in the control group (*M* = 5.88, SD = 0.73 vs. *M* = 3.37, SD = 0.93, *F* = 235.84, *p* < 0.001). Our manipulation in eliciting participants in imagining themselves working with a humble leader was effective. The participants placed in the humble leadership group (*n* = 52) rated work-related promotion focus as significantly higher compared to those placed in the control group (*n* = 52). We conducted a regression analysis to test H1. Results supported that the effect was positive and significant (β = 0.63, *p* < 0.0001; *R*^2^ = 0.39, *p* < 0.0001). To test H2 and H3, we conducted a bias-corrected bootstrap analysis (1000 samples) with Mplus 7.2. Results indicated that humble leadership had a positive and significant indirect influence on employee resilience via increased work-related promotion focus (indirect effect = 0.11, CI 99%, [0.04, 0.24], *p* < 0.01) and exerted a positive and significant indirect effect on employee resilience via enhanced perceived insider identity (indirect effect = 0.12, CI 95%, [0.004, 0.25], *p* < 0.1). All of these effects (direct and indirect) are presented in Table 4.

**Table 4:** Standardized direct path coefficients of the hypothesized model (study 2).

	Path	Estimate	SE		
H1	HL-ER	0.63***	0.07		
**Bootstrap results for indirect effects**		
	**Path**	**Estimate**	**SE**	**LL99%CI**	**UL99%CI**

H2	HL-WPF-ER	0.11**	0.04	0.04	0.24
				**LL95%CI**	**UL95%CI**
H3	HL-II-ER	0.12+	0.06	0.004	0.25

*N = 104. Bootstrap sample size = 1000. ^∗∗^*p* < 0.01, ^+^*p* < 0.1*.

## Discussion and Implications

Study 1 examined the effects of humble leadership on employee resilience through multiple pathways. As hypothesized, both main and heterogeneous effects exist. The findings suggest that humble leadership increased employee work-related promotion focus and perceived insider identity, which in turn resulted in greater employee resilience, supporting our hypotheses. We then carried out an experimental study (Study 2) to empirically test the hypotheses by manipulating humble leadership, in an effort to increase internal validity. Study 2 replicated the results of Study 1. We combined a field study with an experimental study, which provided us with the advantages of both methods, with a consequent increase in both the generalizability and internal validity of our research (Zhu and Kou, 2014; Rego et al., 2017).

Our research makes several theoretical contributions. First, our inquiry helps fill the theoretical gap related to the scarcity of studies about the situational stimuli of employee resilience in the workplace. So far, very little is known about the relationship between leadership and employee resilience. The present research provides evidence regarding the power of humble leadership in facilitating employee resilience. Furthermore, the current results reinforce the contagious nature of humility within the Eastern cultural context and empirically confirm that the virtue of humble leadership is an underpinning for developing employees’ strengths. This finding illuminate’s leadership as a new activator of employee resilience that can be developed. This can be achieved by increasing appreciation of employees’ contributions and exhibiting openness to new ideas, which goes beyond traditional resilience-building channels (Bardoel et al., 2014; Britt et al., 2016; Rego et al., 2017). Second, we advance a better understanding of employee resilience, as the current research is a first attempt to explore the underlying mechanism of how employee resilience is activated by humble leadership. Exploring this mechanism is a vital part of theory development that helps scholars understand why a phenomenon occurs. As a socially enacted and embedded phenomenon (Powley, 2009), the process of employee resilience activation that was tested suggests that intrinsic motivational orientations that leader elicits among employees may exploit a dual pathway of how humble leadership relates to employee resilience. We found that, by displaying an objective self-evaluation, showing openness to new ideas, and showing appreciation to employees’ thoughts, humble leadership facilitated employees’ work-related promotion focus, as well as their perceived insider identity in work organizations, allowing them to further develop resilience.

Our findings also provide some managerial implications. First, our research shows the impact of humble leadership on catalyzing employee resilience in work organizations. Employee resilience can be facilitated in any work environment (Tonkin et al., 2018). Understanding the influence of leader behaviors in the development of employee resilience will advance the development of resilience training programs (Nguyen et al., 2016; Tonkin et al., 2018). Humility, in particular, can be learned and developed (Argandoña, 2015; Rego et al., 2016, 2017; Wang et al., 2018b). Leadership training programs that emphasize nurturing appropriate self-reflection can help facilitate a positive state of development for employee resilience. Leaders should consider enacting humble behaviors, such as appreciating employees’ strengths and emphasizing employees’ developmental journeys (Owens and Hekman, 2012) in order to promote employee resilience. Second, our research finds that both work-related promotions focus and perceived insider identity mediate the relationship between humble leadership and employee resilience. Thus, organizations should pay more attention to developing employees’ work-related promotion focus and creating conditions where employees feel very much a part of their work organizations. To achieve this, our research suggests that some actions taken by the leader, such as giving reality-based feedback, highlighting employees’ strengths and contributions, as well as admitting leaders’ own limits and past mistakes, are useful for facilitation of these two internal motivators (work-related promotion focus and perceived insider identity) of employee resilience.

## Limitations and Future Directions

Despite its strengths, our research has potential limitations that offer promising directions for future research. The first limitation is rooted in the cultural context within which both studies were conducted. Humility is culturally bound (Rego et al., 2017). Although Asian culture has a stronger natural inclination toward humility (Ou et al., 2015), and humility has been considered more important for effective leadership in Asia (Oc et al., 2015; Rego et al., 2017), we recommend that future research be conducted to test the generalizability of the current results in other cultures. Second, Study 1 used a self-report measure that may entail the problem of CMV. As mentioned by Spector (2006), CMV concerns that are associated with heavy reliance on self-reported data measurements may be overstated. However, the current results showed that CMV was not a serious problem in the field study. In order to further avoid this potential problem, an experimental study (Study 2) was conducted to corroborate what we found in the field study (Study 1). We admit that whether there was response bias in the participants’ ratings remains a question. Therefore, we encourage future research to adopt objective measures to replicate our findings and to strengthen causal inference.

## Author Contributions

All authors listed have made a substantial, direct and intellectual contribution to the work, and approved it for publication.

## Conflict of Interest Statement

The authors declare that the research was conducted in the absence of any commercial or financial relationships that could be construed as a potential conflict of interest.
